# Prevalence of atrial fibrillation and reasons for undertreatment with oral anticoagulants

**DOI:** 10.1007/s11239-023-02890-y

**Published:** 2023-09-13

**Authors:** Johan Lilja, Anders Själander, Sara Själander

**Affiliations:** 1 Department of Public Health and Clinical Medicine, Umeå University, Umeå, Sweden; 2 Department of Cardiology, Sundsvall Hospital, 856 43, Sundsvall, Sweden

**Keywords:** Atrial fibrillation, Prevalence, Oral anticoagulation

## Abstract

**Objectives:**

To investigate the prevalence of atrial fibrillation (AF), the proportion of AF patients not receiving oral anticoagulation (OAC) and reasons for abstaining from OAC treatment.

**Methods:**

A retrospective cross-sectional study of patients aged 18 years or older with an AF diagnosis on June 1st 2020 in Västernorrland County, Sweden. AF diagnosis was retrieved using the ICD10 code I.48, and medical records were reviewed for comorbidities and documented reasons to abstain OAC treatment.

**Results:**

Of 197 274 residents in Västernorrland County, 4.7% (9 304/197 274) had a documented AF diagnosis. Of these, 19% (1 768/9 304) had no OAC treatment, including 4.2% (393/9 304) with no indication, 2.5% (233/9 304) with a questionable and 2.5% (231/9 304) with a documented clear contraindication for OAC. In total 9.8% (911/9 304) were not treated with OAC despite indication and no reasonable documented contraindication, thus 90.8% (8 447/9 304) of all AF-patients were eligible for OAC treatment. Common reasons for abstaining treatment without reasonable contraindication were present sinus rhythm in 13.7% (125/911), perceived not an OAC candidate in 10.6% (97/911) and anemia in the past in 4.3% (39/911).

**Conclusions:**

In the population of Västernorrland County, a very high AF prevalence of 4.7% was found, of which just over 90% would theoretically benefit from OAC treatment. This is higher than previously reported and stresses the importance of stroke prevention in this large patient group.

## Highlights

Atrial fibrillation prevalence is 4,7% among adults in Västernorrland county in northern Sweden.

In patients with atrial fibrillation, 9,8% were without oral anticoagulation despite indication and no known contraindication.

For a large proportion of patients, reasons for abstaining oral anticoagulation were not documented in the medical records.

In this atrial fibrillation population, about 90% of the patients could be eligible for oral anticoagulation.

## Introduction

Atrial fibrillation (AF) is the most common cardiac rhythm disorder and confers an increased risk of stroke [1]. AF is reported to affect approximately 3% of the Swedish population [2] [3], a prevalence figure that does not include AF patients treated in primary care only. Oral anticoagulants (OAC) are of great importance in AF to prevent embolic stroke [4–8]. The risk of stroke and guidance on OAC treatment in AF patients can be assessed using different schemes, of which CHA_2_DS_2_-VASc (Congestive heart failure-1p, Hypertension-1p, Age 75 years or older-2p, Diabetes mellitus-1p, previous Stroke/TIA/thromboembolism-2p, Vascular disease-1p, Age 65–74 years-1p, Sex category (female)-1p) is currently recommended [9]. OAC treatment is indicated when the score is two or above for men or three or above for women and should be considered in men with a score of one and in women with a score of two [9]. Swedish national guidelines state that 80% of patients with AF should receive stroke prophylaxis [10].

Undertreatment with OAC is common, leading to ischemic strokes that could have been prevented [11–15]. Despite higher stroke risk in the elderly, the use of OAC is decreasing with increasing age [2, 11, 12]. Acetylsalicylic acid (ASA) is no longer indicated as stroke prophylaxis in patients with AF [9], but might still be perceived as a milder treatment option to frail elderly [2]. Since the introduction of New Oral Anticoagulants (NOAC), the use of ASA has decreased, but there is still undertreatment with OAC in patients with high stroke risk [13, 14]. Also, women with AF are less likely to receive OAC treatment compared to men [2, 13], despite having higher risk of stroke.

Reasons for poor adherence to guidelines have not been extensively studied, but some reasons have been proposed concerning perceptions and attitudes of patients and physicians. Among these are: non-compliance among patients, doctors not paying attention to or doubting guidelines, feeling of uncertainty in making individualized treatment decisions based upon risk-benefit assessment, fear of delegating responsibility for bleeding complications and doctors getting biased thinking patients are going to refuse treatment due to risk of bleeding [12, 16–20].

Studies have previously shown that reasons for abstaining treatment with warfarin in AF patients are insufficiently documented in the medical records. Among documented reasons, risk of falls and other factors related to an increased risk of bleeding as well as sinus rhythm in AF patients were noted [21]. It is common, but not always clinically motivated to discontinue treatment with OAC [22]. Risk of falls is for example usually not a clinically motivated reason for not to treat with OAC in atrial fibrillation patients [23].

The aim of this study was to investigate the prevalence of AF, the proportion AF patients not receiving OAC as well as reasons for abstaining OAC treatment.

## Materials and methods

The total population ≥ 18 years old in Västernorrland County, Sweden on June 1st, 2020, was retrieved from Statistics Sweden, in total 197 274 persons [24].

Digital medical records were used to identify all patients with a diagnosis of AF in Västernorrland County, both in primary and specialized care. AF was defined as an ICD-10 (International Classification of Diseases) diagnostic code of I.48 before June 1st 2020. Patients deceased before June 1st 2020 were excluded, as well as patients registered in another county.

For data analysis, Excel 2020 (Microsoft Corp., Redmond WA) was used. For background information according to CHA_2_DS_2_-VASc in patients without OAC treatment as well as possible contraindications for OAC treatment, medical records were reviewed. Reasons for abstaining OAC treatment were categorized into: (1) Patients without indication for OAC, patients with a contraindication for OAC or patients with a reasonable reason to abstain from OAC treatment, or (2) Patients with indication for OAC treatment and no reasonable reason to abstain from OAC. The categorization is shown in Table 1.

**Table 1:** Categorization of reasons for not using oral anticoagulation treatment (OAC)

Reasonable reasons to abstein from OAC	Doubtful reasons to abstein from OAC
No indication according to CHA2DS2-VASc	Ongoing sinus rhythm
Bleeding	Anemia in the past
Severe kidney or liver failure	Minor risk of bleeding (mild elevated liver enzymes, easy bruising)
Excessive alcohol use	Risk of falls
Atrial appendage plug	A trigger which provoked AF
Poor compliance	No symptoms of AF
Cognitive impairment	First or short AF episode
Patient’s demand	Old age
	Other, including mental retardation

The research project and data collection were approved by the Swedish Ethical Review Authority, registration number 2020 − 01924.

## Results

### Study population

A diagnosis of AF was found in 9 922 patients in electronic medical records from both primary and specialized care. Patients deceased before June 1st, 2020 (n = 399) and patients registered in another county (n = 9) were excluded from the analysis. Of the remaining 9 514 patients, 7 536 were on OAC treatment on June 1st, 2020. Out of 1 978 untreated patients, 210 patients with an incorrect AF diagnosis (misinterpreted electrocardiogram) were excluded, resulting in a final cohort of 9 304 patients with a verified AF diagnosis of which 19% (1 768/9 304) had no OAC treatment (supplementary Fig. 1).

**Fig. 1:**
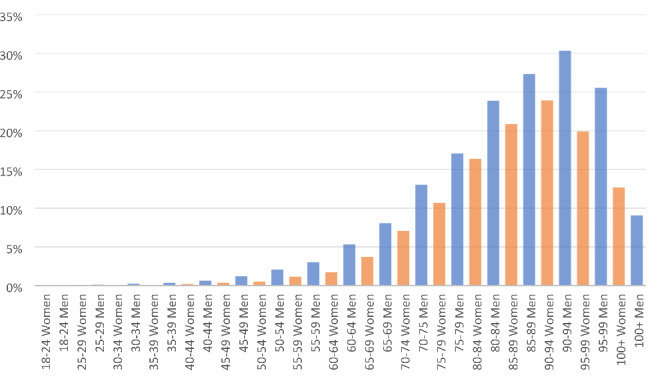
Prevalence of atrial fibrillation in relation to age and gender in the adult population in Västernorrland County, June 1-2020

The total population aged 18 years or older in Västernorrland County was 197 274 [24], giving an overall AF prevalence of 4.7% in the adult population. The highest prevalence, 30.3%, was found in men aged 90–94 years (Fig. 1).

OAC treatment was prescribed to 81% (7 536/9 304) of the patients, 2.9% (270/9 304) were treated with ASA only (Table 2). In total 90.8% (8 447/9 304) of all AF-patients were eligible for OAC treatment.

**Table 2:** Characteristics of patients with atrial fibrillation without OAC treatment, n (%)

Baseline variables	Total: n = 1768 n (%)	Undertreated: n = 911 n (%)
Sex Female	677 (38.3)	377 (41.4)
Age (y) Mean (SD) < 65 65–74 $\ge$ 75	71.1 (15.9) 585 (33.1) 347 (19.6) 836 (47.3)	75.5 (12.2) 144 (15.8) 246 (27.0) 521 (57.2)
Hypertension	1079 (61.0)	704 (77.3)
Chronic heart failure	274 (15.5)	145 (15.9)
Diabetes	287 (16.2)	184 (20.2)
Stroke/TIA	217 (12.3)	112 (12.3)
Vascular disease	359 (20.3)	243 (26.7)
Antiplatlet drugs ASA ADP-inhibitors NSAID DAPT	508 (28.7) 392 (22.2) 62 (3.5) 32 (1.8) 22 (1.2)	344 (37.8) 270 (29.6) 45 (4.9) 12 (1.3) 17 (1.9)
Bleeding Intracranial Gastrointestinal Other	127 (7.2) 50 (2.8) 55 (3.1) 22 (1.2)	0 (0)
Renal failure	28 (1.6)	0 (0)
Liver failure	16 (0.9)	0 (0)
Anemia	372 (21.0)	190 (20.9)
Cognitive impairment	156 (8.8)	0 (0)
Excessive alcohol use	72 (4.1)	0 (0)
Previous OAC treatment Warfarin NOAC Both	695 (39.3) 389 (22.0) 227 (12.8) 79 (4.5)	321 (35.2) 201 (22.0) 96 (10.5) 24 (2.6)
Last seen by a doctor (days/mean)		
CHA2DS2-VASc Mean (SD) 0 1 2 3 4 5 6 7 8+	2.9 (2.1) 286 (16.2) 266 (15.0) 202 (11.4) 262 (14.8) 334 (18.9) 223 (12.6) 116 (6.6) 60 (3.4) 19 (1.0)	3.5 (1.7) 0 (0) 127 (13.9) 160 (17.6) 165 (18.1) 217 (23.8) 136 (14.9) 64 (7.0) 26 (2.9) 13 (1.4)
HAS-BLED Mean (SD) 0–2 $\ge$3	2.0 (1.5) 1055 (59.7) 713 (40.3)	2.3 (1.1) 521 (57.2) 390 (42.8)

### Patients without OAC treatment

AF patients without OAC treatment (n = 1 768) had a mean age of 71.1 years and 38.3% (677/1 768) were women. The mean CHA_2_DS_2_-VASc and HAS-BLED score were 2.9 and 2.0, respectively (Table 2).

Low risk for AF-related stroke was found in 286 men with a CHA_2_DS_2_-VASc score of zero and 107 women with a CHA_2_DS_2_-VASc score of one, which means 4.2% (393/9 304) had no indication for OAC treatment. Contraindications for OAC treatment were found in 2.5% (231/9 304), including bleeding, excessive alcohol use, kidney and liver failure. In total 6.7% (624/9 304) were considered to have a reasonable reason to abstain OAC treatment (supplementary Fig. 2). In addition, 2.5% (233/9 304) had a reason for abstaining treatment that was probably reasonable, of which: cognitive impairment 49.8% (116/233), patient’s demand 48.9% (114/233) and poor compliance 1.3% (3/233).

Undertreatment, defined as not on OAC treatment despite indication and no documented appropriate contraindication was found in 9.8% (911/9 304). In this group, 41.4% (377/911) were women and the average age was 75.0 years. The mean CHA_2_DS_2_-VASc and HAS-BLED score were 3.4 and 2.2. In this group, 50 women and 123 men had a more questionable indication for OAC with a CHA_2_DS_2_-VASc score of 2p and 1p, respectively.

**Fig. 2:**
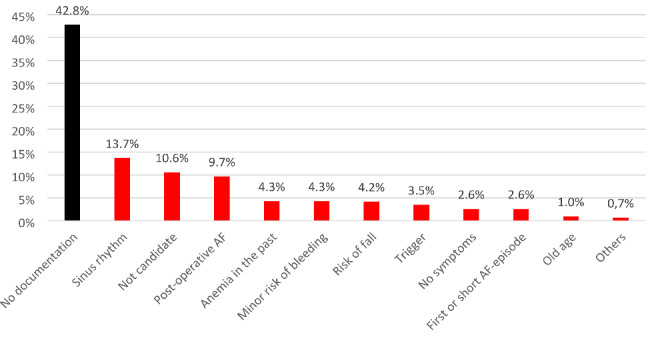
Non-appropriate reasons of no OAC from medical records, n = 911

**Fig. 3:**
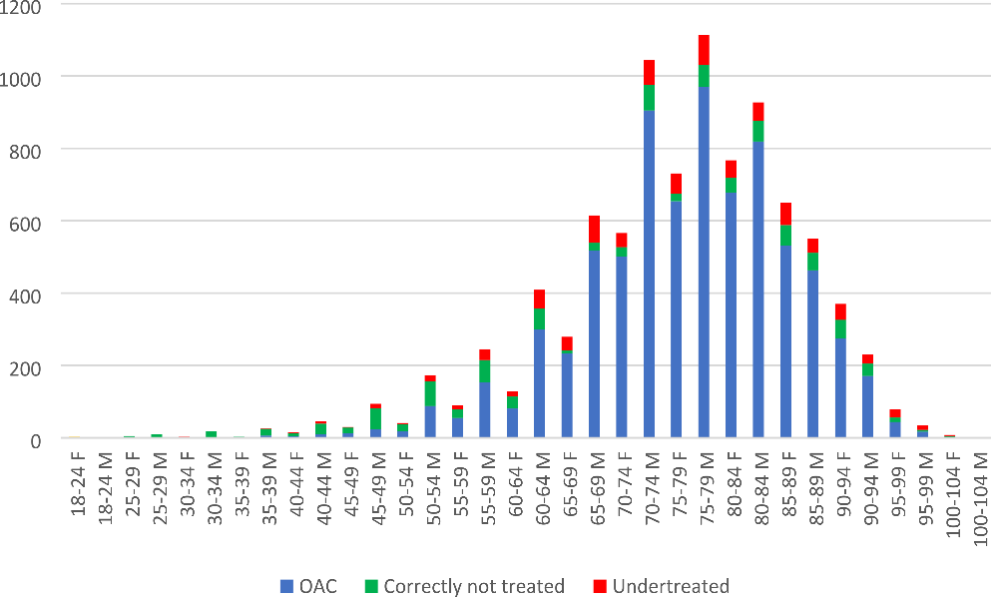
Numbers of AF patients with and without OAC treatment in Västernorrland County divided by age and gender

Documented reasons of why no OAC treatment was prescribed were present in the medical record in 57.2% (521/911) of the patients. The most common documented reason was current sinus rhythm in 13.7% (125/911), followed by that the physician did not see the patient as an OAC candidate in 10.6% (97/911) and post-operative AF in 9.7% (88/911) (Fig. 2). Undertreated patients were seen in almost all age groups and in both sexes (Fig. 3).

## Discussion

The prevalence of AF in Västernorrland County, Sweden was 4.7%, which is higher than previously reported [2, 3]. One explanation to the high AF prevalence is the high mean age in the study population of 43.8 years compared to 41.3 years overall in Sweden (Statistics Sweden, Population statistics 2019). In a study by Friberg et al. [2], the AF prevalence ranged from 2,5% in the Swedish capital Stockholm where the mean age was 39.0 years, up to 3.5% in Norrbotten County in the very north of Sweden, where the mean age of the population was higher, 42.4 years. However, they retrieved the AF-diagnosis from national health care registers, in which only specialized care is available. We here report data from computerized medical records, including AF patients from both primary and specialized care. Our higher AF prevalence partly depends on also including AF patients followed in primary care only.

More than 80% of the AF patients were on OAC in this study with the theoretical possibility to reach 90% when treating all AF patients with indication and no contraindication for OAC. There is a theoretical possibility that some of the patients on OAC also had a contraindication, but the goal for OAC treatment should still be close to 90% of the AF population.

OAC treatment confers both risks and major benefits for the patients, and the clinical decision behind prescribing OAC or not should preferably be documented in the medical records. However, in almost half of the undertreated patients such documentation was missing, in line with previous studies [21]. When reasons for not prescribing OAC were present, these reasons included current sinus rhythm, risk of falls and that the patient was considered “not to be a candidate” (not further specified) for NOAC treatment.

Previous studies have shown that current sinus rhythm is a common cause (16-33.9% of untreated AF patients with indication for OAC) to abstain from OAC treatment in patients with AF [20, 21]. In our study, 15.2% of untreated patients had current sinus rhythm as the documented reason. Despite new OAC available and current guidelines stating that the clinical pattern of AF should not condition the indication for OAC treatment [9], there is still a perception among many physicians that current sinus rhythm reflects a low stroke risk.

Among undertreated AF patients, 4.6% were considered to have too high bleeding risk due to risk of falls, which is lower than previously reported on warfarin (6.4–39.5%) [21, 22]. It has previously been reported that a patient must fall many times for anticoagulation therapy to be contraindicated [23]. This is nowadays more known among the physicians, leading to “risk of falls” being a less common and hopefully decreasing reason to abstain from OAC treatment in AF patients in Västernorrland County.

Women were undertreated with OAC more often compared with men, which was also found by Friberg et al. [2]. Almost 10 years have passed between these two studies, but no improvement in treatment practice was found, despite the higher stroke risk in women [25].

Among all AF patients, 2.4% (224/9 304) were treated with ASA only. This is a historically low figure, the overuse of ASA among AF patients shown previously, especially among the elderly, is at a considerably higher levels [13, 15]. The introduction of NOACs and clear guidelines since several years might finally have proven effective. ASA may be perceived as a milder treatment option afflicted with lower bleeding risk but has in fact similar bleeding risk as apixaban therapy [26]. The indication for aspirin treatment could be another diagnosis than AF, but in patients with for example chronic coronary syndrome and AF, OAC is recommended rather than antiplatelet therapy [9].

### Limitations

We did not verify the diagnosis of AF in patients with OAC treatment like we did in untreated patients. Since the treated patients had a registered diagnosis of I.48 according to ICD-10 in the medical record, there was a physician behind the decision to treat and therefore confirm the AF diagnosis.

## Conclusions

We here report a high AF prevalence of 4.7% in the population of Västernorrland county in Sweden. Undertreatment with OAC is common, especially in women, and reasons for abstaining OAC were not always present or appropriate. Since OAC is crucial in these patients, the decision to prescribe OAC or not is essential to document, and appropriate reasons for abstaining OAC treatment need to be further disseminated among the physicians through local as well as national treatment guidelines. Optimally up to 90% of the patients could benefit from stroke prophylaxis with OAC.

## Data Availability

Because of the sensitive nature of the data that support the findings of this study, data cannot be shared publicly for ethical reasons. Data is available from the corresponding author on reasonable request.

## Declarations

Anders Själander was the principal investigator of the RE-VERSE AD study in Sweden and has received consultancy or lecture fees from Bayer, Boehringer-Ingelheim, Bristol-Myers Squibb, Merck Sharp and Dohme, Portola and Pfizer.

Sara Själander has received consultancy or lecture fees from Bayer, Bristol Myers Squibb and Pfizer.
